# Visual simulation of intraocular lenses: technologies and applications [Invited]

**DOI:** 10.1364/BOE.546971

**Published:** 2025-02-13

**Authors:** Susana Marcos, Pablo Artal, Linda Lundström, Geunyoung Yoon

**Affiliations:** 1 Center for Visual Science, The Institute of Optics, Flaum Eye Institute. University of Rochester, New York, USA; 2 Laboratorio de Optica, Universidad de Murcia, Spain; 3 Department of Applied Physics, KTH - Royal Institute of Technology, Stockholm, Sweden; 4 University of Houston College of Optometry, Houston, Texas, USA

## Abstract

Cataract surgery requires selecting an intraocular lens (IOL), whose design affects visual outcomes. Traditional IOL evaluation relies on optical models and bench testing, but these methods fall short in simulating perceptual factors crucial to patient experience. Visual simulators, based on different principles including adaptive optics, temporal multiplexing or physical projection of the IOLs, now allow patients and clinicians to preview and compare different IOL designs preoperatively. By simulating real-world interactions of the eye’s optics and the visual system with IOLs, these simulators enhance the patient decision-making process, enable personalized cataract surgery, and can aid in regulatory assessments of IOLs by incorporating pre-operative patient-reported visual outcomes. Visual simulators incorporate deformable mirrors, spatial light modulators and optotunable lenses as dynamic elements to simulate monofocal, multifocal and extended depth-of-focus IOLs, including newer designs aimed at improving contrast sensitivity, expanding depth of focus, and minimizing visual disturbances. With ongoing advancements, these simulators hold potential for transforming IOL design, regulatory processes, and patient care by providing realistic and patient-centered visual assessments, ultimately leading to more successful, individualized surgical outcomes.

## 1. Introduction

Cataract surgery, one of the most common surgical procedures worldwide, involves the removal of the opacified natural crystalline lens in the eye and its replacement with an artificial intraocular lens (IOL). The choice of IOL is crucial as it impacts postoperative visual outcomes, patient satisfaction, and quality of life.

The type of lens is a fundamental issue in the process, with new designs being available continuously. The design process of an IOL involves optical simulations in computer eye models, which generally are simplified optical models based on the geometry and anatomy of the eye producing rough estimates of the optical quality metrics. Once the physical lens is manufactured, this is normally tested in an optical bench, using physical model eyes with an either aberration-free cornea or a cornea with positive spherical aberration, which allows predictions of the Modulation Transfer Function and degradation of target images on the retina. While computer eye models and on bench testing provide insights into the prospective optical performance of the IOL designs, they generally fail at capturing the complex interactions between the optics of the eye and the lens design, and more importantly, they do not include perceptual aspects that are critical to individual’s visual experience.

Visual simulators of IOLs are advanced optical and computational instruments designed to aid ophthalmologists and patients in the preoperative assessment and selection of the most suitable IOL for cataract surgery or refractive lens exchange [1]. Furthermore, in those visual simulators in which the lenses are programmed as an active or passive element such as a deformable mirror, a spatial light modulator (SLM), an optotunable lens or a phase plate, they allow a subject to experience a given IOL design prior to effectively manufacturing it. Visual simulators are therefore useful tools when designing IOLs as they provide visual feedback early in the process, even before producing the lenses, speeding up the iterative process of IOL development.

In standard clinical practice, the selection of IOLs relies heavily on ocular biometry and on the surgeon's experience. However, the advent of visual simulators has the potential to revolutionize this process by including the patient’s visual experience. Such an approach is complementary to other advanced IOL selection procedures based on ocular anatomy and 3D geometry obtained from quantitative imaging technologies and ray tracing on patient customized eye models [2]. By offering a virtual preview of postoperative vision, visual simulators help patients understand the potential benefits and limitations of various IOL options, such as monofocal, multifocal, extended depth-of-focus (EDOF) and, if using binocular visual simulators, monovision and modified monovision or mix and match IOL implantation. Visual simulators therefore assist surgeons in optimizing surgical outcomes by simulating the IOLs. They enable the simulation of various surgical scenarios, like varying the IOL model or fine-tuning spherical correction. This interactive and educational experience enhances patient involvement in the decision-making process, leading to more informed choices and expectedly higher satisfaction rates. This capability could pave the way for personalized cataract surgery in the future.

Visual simulators could also play a fundamental role in the regulatory processes for IOL approval by bridging the gap between applications to improve IOL design and to select the IOL in the clinic. Patient reported outcomes questionnaires have been proved useful instruments to assess symptoms and general perceptions in patients with implanted IOLs both in clinical studies and clinical eye care [3,4]. In fact, the US Food and Drug Administration (FDA) has designated the American Academy of Ophthalmology’s Assessment of IOL Implant Symptoms (AIOLIs) as Medical Device Development Tool (MDDT) to clinically evaluate patient’s reported perception of visual disturbances following IOL cataract surgery with premium IOLs (which comprise primarily multifocal and EDOF IOLs). Given their ability to discriminate symptoms (i.e. visual quality, presence of halos, etc) across different IOL designs, AIOLIS is expected to be a useful tool in clinical trials for approval of new IOLs, in what needs to be nevertheless a post-operative assessment. On the other hand, the Medical Device Consortium, a public-private partnership involving industry, healthcare providers, non-profit organizations and public agencies (such as the FDA) advocates for computer modeling and simulations to become a validated and accepted part of a clinical trial, with the aim of simplifying and accelerating regulatory processes [5]. Undoubtedly, visual simulators of IOLs that allow patients to provide prospective visual outcomes of IOL implantation, and even compare performance of different IOLs, will provide enormous value in clinical trials, harnessing the strengths of the information obtained from patient reported outcomes, but in a pre-operative setting.

In summary, visual simulators of IOLs represent an advancement in ophthalmic care, bridging the gap between IOL design, clinical assessment and patient expectations. Simulators could enhance IOL production, regulatory processes, better-informed decisions, personalized treatment plans, and improved visual outcomes, ultimately enhancing IOL developments, and the overall patient experience in cataract and refractive lens surgery. This paper reviews the state-of-the-art of IOL Visual Simulation technologies, their accuracy in predicting pre-operatively post-operative vision with different IOL designs, and their applications in both IOL development and clinical practice.

## 2. Intraocular lens visual simulators

The most straightforward simulations of IOLs are purely computational, mathematically recreating (using Fourier Optics and convolution operations) the image through the optical system of the IOL. These optical computations, as well as their on-bench counterparts, where images are physically obtained through the IOL, may be useful to illustrate some of the principles of the lens but do not represent the actual perceptual image. Also, when shown to the subject they fail to capture the complex interaction with the aberrations of the eye, and to reproduce the retinal projection of a real IOL. One category of visual simulators uses adaptive optics, a technique developed for astronomy, to manipulate the aberrations of the eye and mimic the IOLs in the form of phase patterns mapped on Deformable Mirrors (DM) or SLM [6–10]. A clear advantage of these simulators is the possibility to program any lens prior to it being manufactured, and rapidly compare across designs. Typically, the AO Visual Simulators (AOVS) are not see-through as they are bound by the reflective nature of the DM/SLM and by the limited field of view. Clearly, clinical applications will benefit from a more natural viewing condition (larger fields of view and see-through) while the patient experiences different IOLs. This is the motivation for the second category of visual simulators, some AO-based [11] and others working under alternative principles such as temporal multiplexing [12], featuring binocular viewing, see-through un-obstructed view of the visual scenes, small (wearable) footprint, and seamless transition across simulated IOLs, making them very suitable to use in the clinic. The third category of visual simulators are also see-through systems, but utilizing physical IOLs inserted in a cuvette (similarly to the on-bench measurement) and projected on the patient’s eyes. They thereby have the ability to mimic all monochromatic and chromatic characteristics of the manufactured IOL.

### 2.1. Adaptive optics visual simulators of IOLs

Adaptive Optics (AO) Visual Simulators [1,10,13–15] rely on the capability of active elements, positioned in a conjugate pupil plane, to manipulate the aberrations of the eye, which are measured using wavefront aberrometers (i.e. Hartmann-Shack wavefront sensor). The aberrations can be corrected in real time (closed loop), typically with a DM, or modified according to the design of an intraocular lens. The phase pattern describing an IOL can be mapped using a DM, if the profiles are smooth, such as those described by varying spherical aberration, or using a SLM, such as for diffractive IOLs or refractive segmented IOLs, with sharp transition zones. It is also feasible to place an actual IOL and a phase plate with the IOL design without power in the pupil conjugate, and test visual performance [16,17].

**Fig. 1.**
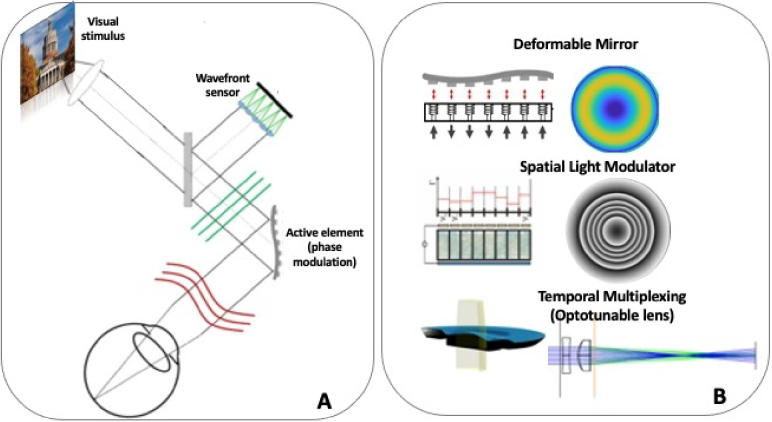
**A.** IOL AO Visual Simulator (AOVS) general concept. IOLs are simulated on a pupil conjugate plane via an active element that reproduces the phase changes introduced by the IOL. **A** Hartmann-Shack (optional) allows measurement of the eye’s aberrations and (if needed) closed-loop correction. Visual stimuli are seen through the simulated correction. **B.** Different active elements found in AOVS: Electromagnetic Deformable mirrors (top), Spatial Light Modulator (middle) and optotunable lens (bottom), separately or simultaneously in the same system.

Since the demonstration of vision improvement (visual acuity and contrast sensitivity) by correction of the high order aberrations of the eye, in seminal work by Liang et al. [18], AOVS have been developed in multiple laboratories and used in fundamental investigations of the spatial limits to vision, such as the effects of aberrations on visual performance, perceived visual quality of natural images, neural adaptation, or binocular summation. The AOVS systems developed by the authors in their labs (IO/UR [9,19], UM [20], UH [21,22], KTH [23]) have been specifically used in IOL research and/or in patients implanted with IOLs, allowing validations of the performance of the simulated IOL with respect to that of the real implanted IOLs. A generic simplified diagram of AO visual simulator is presented in Fig. 1 A.

Table 1 lists specific key elements specific to the different AOVS referred to in the current review.

**Table 1.** Specifications of key components of Adaptive Optics Visual Simulators developed in the authors’ labs^*a*^

	**Hartmann-Shack** ** **	**Deformable Mirror** ** **	**Spatial Light Modulator** ** **	**Light Source**	**Visual Stimulus**	**Spherical correction**
IO/UR	HASO-32, 32 × 32, Imagine Eyes	MIRAO, 52 actuators, Imagine Eyes	LCoS-SLM, 1920 × 1080 Holoeye	Supercontinuum Laser Source SCLS-SC400 femtopower 1060 Fianium (IO). Supercontinuum Laser Source Tango VIS-NIR1 Leukos (UR)	Digital Micromirror Device (DMD Texas Instruments -illuminated by visible light from SCLS through holographic diffusers)	Optotunable Lens EL-10-30-TC Optotune
KTH	HASO4 FIRST, 44 × 36 Imagine Eyes	MIRAO, 52 actuators, Imagine Eyes		Fiber-coupled 830 nm laser diode Thorlabs	Calibrated CRT SONY	Spherical and cylindrical lenses in conjugate pupil plane
UH	Custom-developed	ALPAO, 97 actuators, MIRAO, 52 actuators, Imagine Eyes	SLM JD7554 1920 × 1080, Jasper Display SLM-200 1920 × 1200, Santec	Fiber-coupled NIR Superluminescent Diode InPhenix, Qphotonics	LP LightCrafter Display 4710 EVM, DLPDLCR4710EVM-G2 Texas Instruments OLED display, ECX343EN 1920 × 1200 SONY	Motorized Badal Optometers
UM	Custom-developed	-	LCoS-SLM, X10468; 792 × 600 Hamamatsu Photonics	Fiber-coupled NIR Superluminescent Diodes Thorlabs	SVGA + Rev3 XL OLED eMagin	Trial lenses Optometers

^*a*^ IO: Instituto de Optica; UR: University of Rochester; KTH: Royal Institute of Technology; UH: University of Houston; UM: Universidad de Murcia LCoS: Liquid Crystal on Silicon; SLM: Spatial Light Modulator; SCLS: Supercontinuum Laser Source; VIS: Visible; NIR: Near Infrared; DMD: Digital Micromirror; CRT: Cathode Ray Tube; OLED; Organic Light Emitting Diode

Some unique features of the AOVS systems described here include: (1) Polychromatic illumination (IO/UR KTH, UH) and integrated measurement of the longitudinal and transverse chromatic aberration (IO/UR) [24,25]; (2) Binocular configuration, either by replication of the monocular channel with vergence control (UH) [26] or combining both eyes in the SLM and HS sensor (UM) [27]; (3) Both on-axis and off-axis measurement and simulation (KTH) [23]. Also, UM is developing a wearable binocular AOVS [11] and UR is developing a small-footprint see-through binocular dynamic AO simulator and accommodation tracker.

Limitations of AOVS technologies include normally a small field of view, limited by the aperture of the optics, and/or the need to block spurious light different diffractive orders (SLM); chromatic artifacts in white light, arising from the wavelength-dependence of the phase-wrapping process (SLM); limited stroke and spatial resolution (number of actuators) for deformable lens and mirrors, and generally a large footprint.

### 2.2. Simultaneous vision IOL visual simulators

**Fig. 2.**
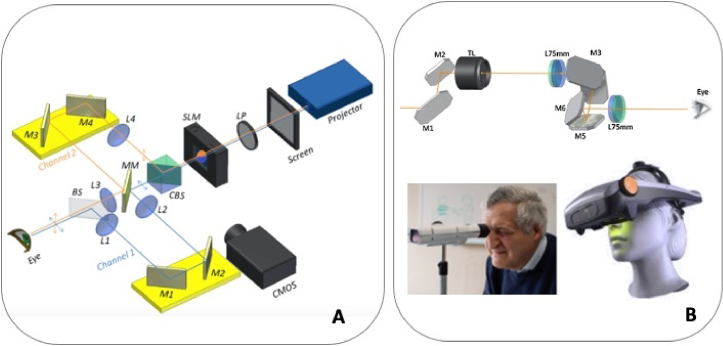
**A.** Two-channel see-through Simultaneous Vision Simulator of Bifocal IOLs. A transmissive SLM allows mapping IOLs with different zonal distribution of near and far. Badal optometers in the two channels allow variable distance correction and near add. Illustration taken from Dorronsoro et al. [33]. **B**. Portable see-through simulators working under the principle of temporal multiplexing with optotunable lenses. *Upper and bottom left*: diagram and photograph representing the system in a monocular configuration. From Dorronsoro et al. [28]. *Bottom right*: binocular head-mounted system where each eye can be simulated with a different lens (SimVis Gekko, courtesy of 2EyesVision).

Multifocal IOLs work under the principle of simultaneous vision, where images in focus at different distances are superimposed on the retina. Examples of Simultaneous Vision simulators exploit that principle bypassing the need to use AO mirrors by specifically projecting focused and unfocused images simultaneously on the retina or by rapid variations in focus, beyond the visual system temporal integration (>30 Hz), producing a static appearance in the perceived image [28–30]. A key feature of these systems is the potential for being see-through, as the active components work in transmission rather than reflection [31]. Examples of different realizations of the SimVis technology appear in Fig. 2. Laboratory implementations of a two-channel SimVis system allow simulations of pure bifocal lenses, with varying energy split between near and far vision, and varying near add [32]. The incorporation of a transmissive spatial light modulator, in combination with polarizers, allows splitting the near and far add components across the lens by a black and white pattern mapped in the Spatial Light Modulator allowed simulations of any segmented bifocal design (Fig. 2(A)) [33].

Temporal multiplexing, achieved by optotunable lenses, allows mimicking any IOL by cycling through a set of powers that tailor the through-focus performance of any given IOL (for example, commercially available multifocal refractive, diffractive or EDOF IOLs) [30]. The use of optotunable lenses, projected on the eye’s pupil (Fig. 2(B) upper panel), has allowed reducing the footprint and weight of the system from a monocular system (Fig. 2(B), lower left) to a binocular wearable device [12] with a wide (close to 20 deg) field of view (Fig. 2(B), lower right). Other advantages of the temporal multiplexing principle are a higher tolerance to pupil decentration (which occurs during convergence at near viewing) given that the IOLs are represented temporally and not spatially, and the ability to bypass crystalline lens opacities, as temporal patterns are less affected than a spatial patterns by spatial obstructions. Due to the temporal multiplexing approach, the system does not directly represent spatial pupillary distributions, particularly IOLs with asymmetrical profiles.

### 2.3. Physical IOL projectors

Physical IOL projectors represent a category of see-through visual simulators that incorporate a real IOL within a water-filled cuvette. These devices offer the advantage of encompassing all optical properties of the IOL, including not only the intended multifocality, but also potential side effects such as aberrations and scattering. Consequently, they are particularly suitable for evaluations in the final stages of IOL manufacturing and for comparative analyses of different IOL models.

The Rassow telescope is one of the earliest physical IOL projectors. It gives an optical projection of the IOL onto the entrance pupil plane of the observer’s eye. The +20 D IOL is positioned in a water-filled chamber and forms a 1:1 telescope together with a + 20 D biconvex lens placed at double focal length distance behind it [34]. However, this projection suffers from several drawbacks, including the fact that the IOL in a pseudophakic eye is situated in the pupil plane of the eye, not in the entrance pupil. Notably, the entrance pupil is approximately 13% larger than the pupil aperture [35], resulting in the IOL being imaged into the eye at a smaller size than its actual physical dimensions. Additionally, the vergence of the incident light on the IOL is close to zero and not convergent, as it would be after passing through the cornea. An alternative design, the concave lens Rassow telescope, incorporates a negative lens instead of the biconvex to neutralize the power of the IOL [36]. However, it shares the same limitations as the original Rassow telescope. The VirtIOL commercial device features a + 40 D lens, mimicking the cornea, with the water-filled cuvette containing the +20 D IOL positioned behind it. This setup allows for the imaging of a distant object approximately 15-30 mm from the IOL, with a third component, a + 60 D lens, facilitating the projection of the object onto the pupil plane of the subject [34]. This configuration offers converging light incident on the IOL and shows better performance than the Rassow telescope [34]. Nonetheless, it suffers from incorrect magnification of the IOL onto the pupil plane of the eye. The ACMIT simulator is also a commercial device that employs an artificial eye with a model cornea and the IOL in a cuvette, together with a relay-optics system that projects the image directly onto the retina of the subject [37]. However, specific details regarding the imaging of the IOL remain unspecified.

**Fig. 3.**
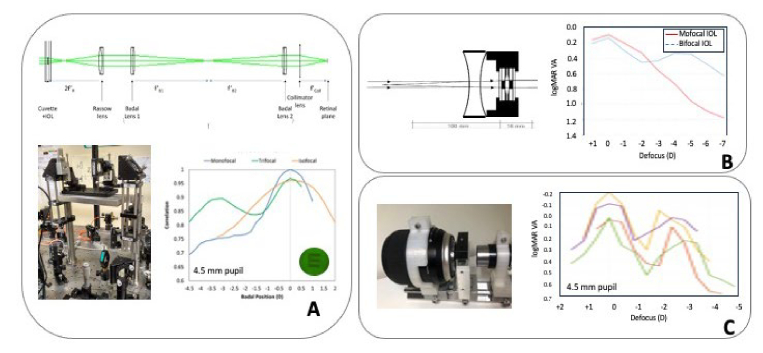
**A.** *Upper panel*: Rassow telescope to project an IOL inserted in a cuvette onto the eye’s pupil. *Lower left panel*: IOL projector module (CSIC Rassow Telescope) incorporated in an Adaptive Optics Visual Simulator (IO); *Lower right panel*: On bench through-focus optical performance of three physical IOLs projected on the pupil plane of an artificial eye in the system. The optical quality metric is the correlation of the image of an E optotype captured in the CCD camera acting as the retina, through the system with no lens, and through a monofocal IOL (blue), trifocal IOL (green), isofocal IOL (orange) inserted in the cuvette. Illustrations are from Benedi-Garcia et al. [38]. **B.** *Right panel*: IOL simulator consisting of a trial lens tube, concave lens, and wet cell where the IOL is inserted. The system is designed to be mounted in a trial frame. *Left panel*: Through-focus visual performance (logMAR VA) with monofocal (red) and bifocal (blue) Tecnics IOLs. Figure adapted from Na et al [36]. **C.** *Right panel*. Photography of KTH IOL telescope. *Left panel*. Through-focus visual performance (logMAR VA) of the same multifocal IOL on four different cyclopeged subjects. From Lundström et al. [40]

The CSIC Rassow telescope is an AOVS that integrates a Rassow telescope within one of its channels. Figure 3 A shows a schematic diagram of the channel (upper panel), and a detail on its integration in the IO AOVS system (lower left). The IOL is positioned in a water-filled cuvette and the telescope projects the IOL onto the entrance pupil plane of the subject [38]. Although the system uses parallel light incident on the IOL, it corrects for the mismatch in magnification of IOL onto the pupil plane. This configuration therefore provides accurate lateral magnification of the IOL diameter onto the physical pupil. The angular magnification differs from one, which may impose some deviation in the scattering transfer, although this has not been detected experimentally. Recent studies have cross-validated results from the CSIC Rassow telescope with simulations achieved by programmable simulators. These experiments paid careful attention to properly compensate for the IOL's base power, as magnification factors associated with the convergence of rays from the cornea to the pupil plane could induce aberrations within the projection system. To avoid this potential artifact the deformable mirror in the system was used as a backup to correct for any present residual aberrations [38]. The cross-validations have been successful for multiple IOL designs [38,39,17] with both refractive and diffractive principles, including monofocal, isofocal, and diffractive trifocal IOLs (Fig. 3(A), lower right panel). Figure 3(B) shows the Na et al [36] IOL projector, designed to be mounted in a trial frame (schematic diagram shown in left panel), and examples of logMAR VA with projections of monofocal and bifocal IOLs (Tecnis family by Johnson and Johnson, right panel).

The most recent advancement in physical IOL projectors is the compact see-through IOL telescope (IOL-T2) developed at KTH (shown in Fig. 3(C), left panel) that transfers all optical properties of the IOL, including scattering, into the pupil plane of the eye [40]. The device features a + 45 mm camera objective paired with a + 20 D IOL in a water cuvette, resulting in an effective power of +40 D to generate an intermediate image after the IOL. This image can then be viewed through a + 40 D eyepiece, thus generating a telescope with an angular magnification of -1. The eyepiece also serves the purpose to, together with the cornea of the observer, project the IOL into the pupil plane, achieving both angular and transverse magnification equal to -1 (provided that the power of the cornea is +40 D). This design achieves both realistic incident vergence onto the IOL and precise projection of the IOL onto the pupil plane in terms of transverse and angular magnification, thereby ensuring phase conservation. The performance of this IOL telescope has been compared to clinical data from different pseudophakic eyes implanted with the same IOL designs. Through-focus visual acuity data (shown in Fig. 3(C), right panel), best-focus contrast sensitivity, and halos closely align with the clinical measurements obtained from patients with implanted IOLs, as well as with theoretical predictions both for monofocal and multifocal IOLs [40]. A drawback, common to the physical IOL projectors is their static nature, limiting the ability to scan through various corrections, such as those digitally programmed in active devices.

## 3. From IOLs to simulated IOL patterns

Programmable Visual Simulators require the IOLs to be mapped as phase maps onto the DM or SLM. Many investigations explore the effect of different phase maps on visual quality at best focus and through-focus. Those phase patterns could eventually be transferred to IOLs, by estimation of the IOL geometry which would result in the corresponding phase aberration. For example, AOVS have been used to assess the impact of, for example spherical aberration [39,41,42], astigmatism and higher order aberrations [22] or combinations of astigmatism and coma on visual quality and DOF expansion [43]. Other studies studied visual performance with lenses with different distributions of near and far across the pupil, simulated in the SLM. While these patterns do not necessarily correspond to existing IOLs, comparative analysis allows previewing optimal phase distribution which can be then transferred into an IOL form [16].

**Fig. 4.**
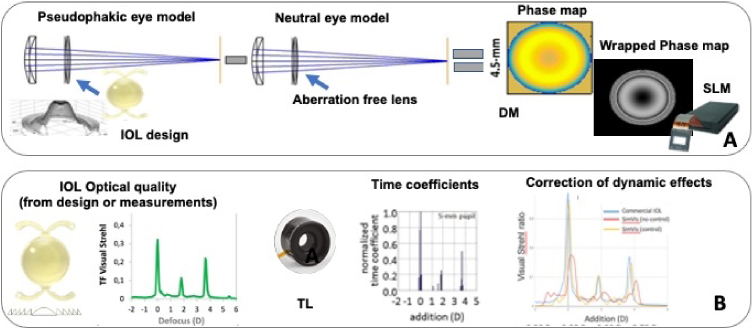
**From IOL design to IOL mapping in visual simulators. A** Estimation of the IOL pupilarly phase pattern by subtraction of the wave aberration in an eye with an aberration- free lens and that of an eye with the phase under test. The phase map representing the IOL can be mapped on the deformable mirror (DM) or as a wrapped phase in a Spatial Light Modulator (SKM). The example corresponds to an Isofocal IOL. The lens profile is from Fernandez et al. [74]. The IOL photograph is from PhysIOL/BVI. Graphics are from Lago et al. [39]. **B.** Estimation of the time coefficients in a temporal multiplexing paradigm with a tunable lens (TL) operating at high frequency. The through-focus (TF) performance of the lens in terms of Visual Strehl or MTF is calculated from the IOL geometrical design or on bench testing, The temporal profile of the lens (time coefficients) is calculated to tailor the lens TF performance. Dynamic effects in the TL are compensated. The example is for a FineVision IOL by BVI-PhysIOL (depicted as picture and as multifocal echelette profile). *Left* and *Middle* graphs are from Akondi et al. [29] and *right* is from Dorronsoro et al. [46]. The TL picture is from Optotune.

Alternatively, the design of a given IOL is transferred to the visual simulator in the form of a phase pattern placed in a pupil plane. There are several alternatives to do that. In one method (used by IO/UR) [39] computer eye models are built with a standard cornea model, one with the test IOL geometry, and the other with a neutral aberration-free lens in place of the crystalline lens/IOL. Wave aberrations are obtained by ray tracing on these two eye models, and the phase pattern representing the test IOL is obtained by subtraction of the wave aberrations of eye with the neutral lens from those of the test IOL (Fig. 4(A)). For diffractive IOLs, the corresponding phase map is obtained directly from the diffractive component of the IOL [17]. Generally, the IOL is only modeled as a phase map, while the amplitude is kept constant (full transmission) within the pupil. A configuration of an SLM in the UH AOVS permits pupil apodization (Gaussian profiles in the pupil transmission function [44]).

For temporal multiplexing, lenses are defined by a set of temporal coefficients that dictate the time fraction that the optotunable lens adopts a certain power, tailoring the through-focus visual Strehl/MTF of any given IOL design [29,30,45]. Mechanical dynamic effects of the optotunable lens [46] and temperature fluctuations [47] are also taken into account to accurately reproduce performance (Fig. 4(B)).

## 4. Validation, demonstrations and applications

### 4.1. Using visual simulators to optimize new IOL designs

**Fig. 5.**
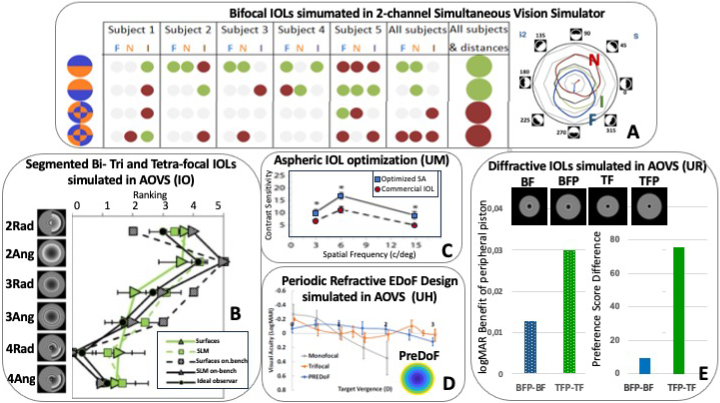
**Testing of novel IOL designs in Visual Simulators. A** Bifocal segmented lenses in a two-channel simultaneous vision simulator. *Left*: Best (green dots) and Worst (red dots) judged patterns by individual subjects (and on average) at Far (F), Intermediate (I) and Near (N). 2-zone angular patterns tend to be preferred by most subjects, although their performance is very orientation-dependent. Hybrid patterns tend to be the most rejected. Results are selected from Dorronsoro et al. [28]. *Right:* Visual quality (scores) for one subject with a 2-Zone angular IOL design at different orientations (270 deg is the optimal orientation at Far for this subject). **B.** Visual ranking at far (scores range:0-5) of bifocal, trifocal and tetrafocal segmented IOL designs, simulated in an AOVS with SLM. Results are average across 5 subjects. For the same number of zones, angular (Ang) outperform radial (Rad) distributions. Quality degrades with increasing number of zones. Data from Vinas et al. [16]. **C.** Contrast Sensitivity in one subject (4.8 mm pupil) measured in white light, for a commercial aspheric IOL which corrects for corneal spherical aberration of the average population (red circles) and one correcting the individual spherical aberration (blue squares). Adapted from Piers et al. [41]. **D.** Periodic Refractive Extended Depth of Focus (PREDoF) IOL design for a 6 mm pupil (right inset). Through-focus logMAR visual acuity tested in a AOVS, with three different profiles mapped on an SLM: PREDoF IOL (blue), a typical monofocal IOL (gray) and diffractive trifocal IOL (orange). Adapted from Lyu et al. [58]. **E.** Visual benefit at far in terms of high contrast logMAR Visual Acuity (left) and visual preference (right) when an annular phase piston is introduced in the periphery of bifocal (blue, 0.25λ piston) or trifocal (green, 0.28λ piston) diffractive IOLs. BF stands for Bifocal, BFP Bifocal with piston, TF Trifocal, TFP Trifocal with piston. Results are selected from Goswami et al. [57]. Designs provided by ClerioVision Inc.

Visual simulators are an excellent platform to test different lens profiles and explore the effect of different design parameters (magnitude near add, number of zones, distributions of areas of near and far, magnitude of spherical aberration, etc) on visual performance. Using a two-channel Simultaneous Vision Simulator, de Gracia et al. [32] investigated systematically the effect of the magnitude of near add in a purely bifocal IOL on high and low contrast visual acuity. Low-medium produced larger degradation than high adds, likely due to the fact that higher amounts of defocus produce a more uniform pedestal than partially defocused images superimposed to a sharp image. The effect was also observed in pure simultaneous vision images perceptual scores, although it was attenuated following brief periods of neural adaptation [48]. Two-channel Simultaneous Vision Simulators provided with a transmissive SLM could simulate segmented IOL designs with concentric, asymmetric and hybrid distributions of equal contribution of far and near (2.5 D). Dorronsoro et al. [28] showed optimal performance with asymmetric patterns (pupil divided into two haves) and the worse performance with highly interleaved regions for far and near (Fig. 5(A), left panel). Notably, these pattern distributions are reminiscent of commercially available IOL designs such as the Lentis MPlus (Teleon, formerly Oculentis) and the Precizon Presbyopic NVA (Ophthec), respectively. Simulation of radial and angular designs with multiple zones in the SLM of an AOVS and perceived visual quality metrics in patients showed that angular designs outperform radial designs, and 2-zone designs perform better than 3-4 zone designs (Fig. 5(B)) [49]. The visual simulators also allowed to investigate the coupling of the optical aberrations of the eye and that of the lens design. For example, the results suggest a patient-dependent optimal orientation of the angular designs (Fig. 5(A), right panel) [50].

The UM AOVS featured one of the first applications of an AO simulator to optimize IOLs. First with the Tecnis IOL (AMO, Santa Ana, CA), an aspheric monofocal IOL aiming at compensating for the average corneal spherical aberration (SA) in a cataract patient population and more recently with the TECNIS Symfony. As SA varies across subjects, residual amounts of SA at the individual level, which could be deleterious to vision but also beneficial by increasing depth-of-focus and protecting from potential focus error. Piers et al. 2004 [41] used the UM AOVS to evaluate the effect of customizing full correction of SA in each individual, finding that SA correction improves vision at best focus (for example contrast sensitivity, see Fig. 5(C)) without significantly affecting the subjective tolerance to defocus. Other studies with this system include improvement of IOLs [51], comparison of different solutions for presbyopia correction [52–55], and identifying the design of IOLs that optimize vision in the peripheral retina [56].

The UH AOVS equipped with an SLM has been used to explore potential improvement of presbyopia-correcting IOLs by generating additional focal points to enhance the continuity of vision across different distances, and in particular a new design that creates four distinct focal points at 0, 1, 2, and 3 D, has been programmed in the system [58]. The results demonstrated improved through-focus visual quality over a 3-D range, with only a minor compromise in overall image quality under monochromatic conditions (Fig. 5(D)). The AOVS allows for further optimization of the design by evaluating diffractive performance and shifts in focal points across a broad spectrum of wavelengths. As an alternative solution, a periodic refractive IOL design was proposed [59]. This design features a power profile that repeats periodically across the lens diameter. The profile can be tailored to adjust the distribution of light energy over a continuous range of object distances and customized to individual patients’ needs. Through-focus visual performance with this design was also assessed using the same SLM-based AOVS, and the results showed that it provides a continuous depth of focus over more than 2D. Additionally, it was found to be more tolerant of variations in pupil size and lens decentration relative to the eye's pupil.

Recent work with the UR AOVS has also shown the use of SLMs to optimize diffractive bifocal and trifocal diffractive IOL designs by modulating peripheral piston in the IOL [57]. A 0.25-0.28λ phase annular peripheral piston improved consistently high and low contrast visual acuity, and vision through these patterns was consistently preferred over similar bifocal and trifocal IOL designs with no peripheral piston (Fig. 5(E)).

### 4.2. Pre-operative simulations of real commercial IOLs match post-operative data

**Fig. 6.**
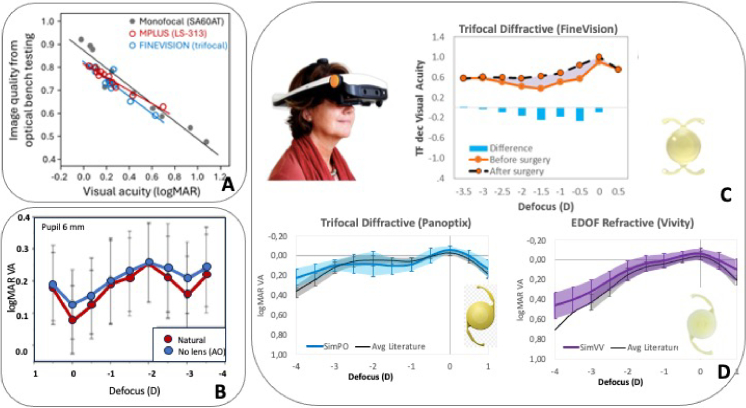
**Pre-operative simulation of post-operative vision with real IOLs A.** Post-operative visual acuity predicted by through-focus retinal image quality of the IOLs (monofocal, Mplus and Finevision) quantified with an optical bench testing system. The plot is adapted from Plaza-Puche et al. [60]). **B .** Through-focus logMAR Visual Acuity through a simulated diffractive trifocal IOL (custom) in patients, with the natural crystalline lens, or the high order aberrations of the crystalline lens neutralized with Adaptive Optics. Data are adapted from Villegas et al. [63] **C.** SimVis simulation of trifocal diffractive IOL (FineVision by BVI-PhysIOL) pre-operatively in comparison with post-operative data in the same patients post-implantation of the IOL. Data are from Vinas et al. [61]. **D.** SimVis simulation of trifocal diffractive (Panoptix by Alcon) (blue lines, left) and of EDOF refractive IOL (Vivity by Acon) (purple lines, right) on presbyopic patients (n = 15) in comparison with post-operative data with these IOLs from the literature. Data are from Zaytoun et al. 2019 [62]**.**

Establishing a relationship between subjective visual performance after implanting a presbyopia-correcting IOL and objective optical quality through-focus is of great clinical importance. A study was conducted to correlate clinical visual performance at various object distances with through-focus optical image quality obtained using a bench testing system [60]. The IOL models tested were the varifocal Lentis Mplus LS-313 (Oculentis GmbH, Germany), the trifocal FineVision (Physiol, Belgium), and the monofocal Acrysof SA60AT (Alcon Laboratories, TX). Significant correlations were found between logMAR visual acuity and cross-correlation coefficients representing image quality across all three IOL models (Fig. 6(A)). This suggests that objective bench testing of an IOL can help clinicians and researchers to predict general visual outcomes following implantation. However, this testing does not incorporate true human visual processing, nor individual responses to the coupling of the eye and IOL optics with the visual system.

The potential role of the aberrations of the crystalline lens on pre-operative simulations was investigated by Villegas et al. [63], who concluded that the effect of crystalline lens aberrations on visual simulation was imperceptible even for large pupil diameters (Fig. 6(B)). Although the presence of lens aberrations introduced subtle differences in VA in some subjects with larger pupil diameters, those were negligible on average, support the use of the pre-operative testing with visual simulators. On the other hand, Barcala et al. [64] demonstrated that rankings in perceived visual quality of a series of presbyopic IOL corrections simulated pre-operatively through cataractous lenses remained unaltered when the same test was performed post-operatively through an implanted monofocal IOL, confirming that the selection of IOLs based on the pre-operatively would correspond with the top choice for the patient.

The ultimate goal of visual simulators is to provide patients with the experience of vision with IOLs non-invasively, allowing a preview of the world through the simulated lenses prior to surgery. For the simulation to be realistic, the simulated lens needs to be a faithful representation of the real IOL. Also, if the simulation is performed on phakic patients (or even patients with cataract) the crystalline lens of the eye needs to play a minor role for the simulation of the post-operative condition (in which the crystalline lens is removed). Using an AOVS provided with a SLM and an optotunable lens working under the principle of temporal multiplexing, Vinas et al [61] showed a large degree of similarity between the through-focus visual acuity pre-operatively with a simulated diffractive trifocal IOL (Fine Vision) and that post-operatively, both with the diffractive lens mapped on a SLM (in form of a spatial pattern, or in the optotunable lens (in a temporal multiplexing (Fig. 6(C)). In another recent study, patients performed through-focus visual acuity measurements through multiple simulated IOLs (FineVision trifocal diffractive IOL by PhysIOL, Panoptix multifocal diffractive IOL by Alcon; Symfony EDOF/diffractive by Johnson and Johnson; diffractive AT Lara and AT Lisa Tri by Zeiss) in a wearable binocular SimVis instrument, and compared them to defocus curves in patients implanted with those IOLs reported in the literature [62]. For the majority of the IOLs the overlap between simulation and the corresponding post-operative curves was 100% (Fig. 6(D)).

### 4.3. Binocular simulations of vision with IOLs: effect of dominance

Cataract surgery is most often performed in both eyes of a patient (generally separated by days or weeks), and therefore simulation of performance of IOLs binocularly will more naturally convey real vision post-operatively. Furthermore, surgeons often intentionally offset the spherical correction between eyes to expand depth-of-focus or use a different lens type for each eye, so called “mix and match” in the IOL implantation jargon. Binocular visual simulators of IOLs are therefore instrumental at providing patients with the experience of different binocular combinations of lenses.

**Fig. 7.**
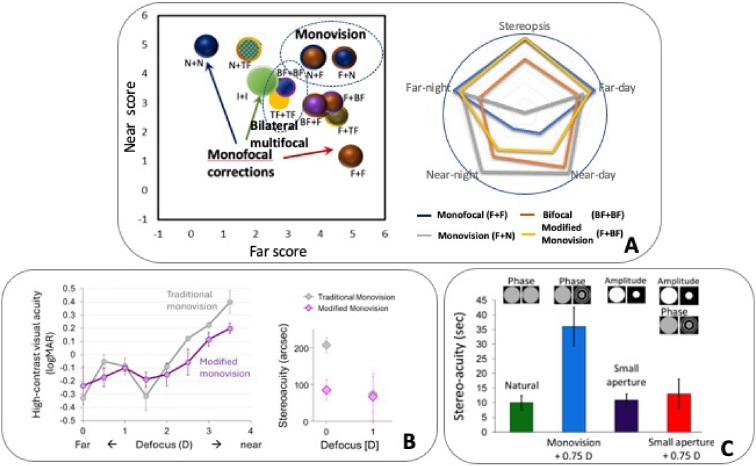
**Binocular Visual simulation of IOLs. A** *Left.* Perceived visual score at far (horizontal axis) and at near (vertical axis) for different combinations of bilateral designs (average across 10 subjects; size of the bubble represents weight of the effect) simulated with a binocular visual simulator. Score ranges from 0 -worse- to 5 -best. F stands for monofocal IOL focused at far; I stands for monofocal IOL focused at Intermediate; N stands for monofocal IOL focused at near; BF stands for bifocal IOL; TF stands for trifocal IOL; X + Y indicates bilateral correction, where X indicates dominant eye and Y indicates non-dominant eye; X = Y indicates equal corrections in both eyes and X≠Y monovision corrections, traditional monovision if X and Y are monofocal IOLs and modified monovision if X and Y are monofocal and BF or TF, respectively, or viceversa. Highest scores of binocular visual quality are found for F + N; F in the dominant eye produces higher scores than N, BF or TF in the dominant eye. Data are compiled from Radhakrishnan et al. [31]. *Right:* Multifocal Acceptance Score polygons for four different combinations of IOLs tested in a presbyopic subject. Scores are for natural images at far, near, and day and night conditions, as well as stereovisual acuity. Values closer to the circle indicate highest quality. Monofocal IOLs (F + F) perform best at far, but low at near, while bifocal IOLs (BF + BF) reduce quality at far at the expense of improving at near. Standard monovision (F + N) produces high quality at far and near, but reduce stereovision. Modified monovision still produced improvement at near without compromising stereo vision in this subject. Result from Barcala et al. [65]. **B**. Through-focus visual acuity (left) and stereoacuity (right) for traditional monovision with +1.5D near add in the non-dominant eye, and modified monovision with a combination of primary and secondary spherical aberrations (+0.1 μm and -0.4 μ m, respectively) for a 4 mm pupil. The binocular AOVS was used for these measurements. Data are adapted from Zheleznyak et al. [42]. **C**. Stereo-acuity with different simulated IOL binocular combinations: natural eye (no IOL), monovision (plano dominant eye and +0.75 D in non-dominant eye), small aperture or small aperture combined with monovision. Figure taken from Fernandez et al. [73]

Perceived visual quality tests using binocular simulators show that flipping the same two different IOL designs (for example a monofocal IOL and a multifocal IOL) between eyes of the same subject results in different perceived judgements [31], as shown in Fig. 7(A) (left panel) in terms of perceptual scores for near and far vision for binocular corrections with different simulated IOL combinations in right and left eye. In certain subjects, standard monovision (where one eye is focused for far and the contralateral eye focused at near) may impair stereo-acuity performance, as shown in Fig. 7(A) (right panel, gray line). In this scoring test, the vertices represent the perceptual scores of natural images at far, near, daylight and low light condition. In the example presented in Fig. 7(A) (right) modified monovision (monofocal IOL in the dominant eye and bifocal IOL in the non-dominant eye appears to be a good compromise [65]. Results shown Fig. 7(B) show that a modified monovision outperforms traditional monovision, particularly for larger amounts of defocus offset between eyes.

Choosing the eye dominant that matters for a monovision prescription is therefore critical for optimal vision, yet the standard practice uses a quick test based on gaze (hole in the card) to determine dominance. However, it is not clear that motor dominance relates to monovision performance. Recent work with binocular simultaneous vision simulators has shown that they can be used to measure monovision preference, identifying not only the dominant eye (which needs to be corrected at far) but also the strength of the dominance [66,67]. The test is performed in a two-alternative choice method by testing perceived quality preference between a randomly presented combination of right and left eye for far and near and reversed. Strong eye dominance will be indicated by a systematic choice of a given combination, while equi-dominance by a random selection of preference.

Using the UH AO Binocular Visual Simulation (BAOVS), Zheleznyak et al. [68] employed sensory dominance tests [69], which measure the imbalance in sensory inputs between the two eyes, in particular a psychophysical paradigm based on binocular rivalry, where each eye is dichotically presented with orthogonally oriented sinusoidal gratings and the degree of dominance is determined by the proportion of time one eye dominates over the other. The results showed that sensory eye dominance significantly affected through-focus contrast sensitivity. Binocular contrast sensitivity was higher when the dominant eye had better optical quality than the non-dominant eye. Interestingly, high contrast visual acuity remained unaffected by either monovision correction strategy.

A challenge of binocular visual simulators is the variability of inter-pupillary distance and eye convergence, and continuously maintain the centration of the simulated IOL centered in both eyes. The studies mentioned above circumvent this problem by simulating the IOL by temporal multiplexing (not relying on a spatial representation) or by simulating only defocus in a standard monovision correction, which is spatially invariant across the pupil.

The BAOVS at UH was developed using two identical monocular AOVS system [26]. The system features custom-developed wavefront sensors and DMs with a large dynamic range, allowing control over the optical quality of both normal and highly aberrated eyes. It also includes custom-designed control software that provides versatility not only correcting the eyes’ aberrations, but also inducing specific aberration profiles. Various vision tests are performed with digital micromirror display using DLP technology (Texas Instruments) while optical manipulation occurs in real time, under both monochromatic and natural white light conditions. Also, vergence control is particularly important for binocular through-focus visual performance testing in non-cycloplegic conditions [70]. In an application of this system, conventional monovision (where only the interocular defocus is modified) was replaced by modified monovision technique in which positive and negative spherical aberrations (SA) of different magnitudes were applied to each eye. Compared to traditional monovision, modified monovision with SA improved through-focus visual acuity and extended depth of focus (Fig. 7(B)). A significant increase in binocular summation was also observed, likely due to the enhanced interocular similarity in image quality resulting from the extended monocular depth of focus. BAOVS offers a powerful tool to further optimize binocular depth of focus by testing various combinations of primary and higher-order spherical aberrations with different signs and magnitudes for each eye. UM proposed an alternative BAOVS approach, first introduced by Fernandez et al. in 2009 [71], in which rather than duplicating the main elements of the AOVS, a LCoS-SLM is adapted to address both eye’s pupils simultaneously. Given the pixelated structure of the corrector device, it is possible to simulate independent corrections for each eye, dividing the active surface for each pupil, with sufficient resolution and without increasing control complexity, while reducing cost and gaining simplicity. This system has been used to study stereopsis, in particular the effects of higher order aberrations [72], monovision and small apertures on stereovision [73]. While stereopsis is degraded by traditional monovision, monovision in combination with a monocular small aperture returns stereopsis values similar to natural viewing.

## 5. Future of IOL visual simulations

Adaptive Optics Visual Simulators have mostly been developed in laboratory environments. However, the versatile applications in the field of IOL development such as guiding the regulatory process, providing pre-operative visual experience and advanced optical metrology are merging the technology into commercial products.

The future of IOL simulators is necessarily linked to the development of technologies that reproduce the refractive or diffractive nature of the IOLs and can capture the optical performance as well as dysphotopsia and chromatic behavior without introducing artifacts. See-through devices allowed by transmissive, rather than reflective active elements are preferred, as they facilitate an unobstructed view of the environment. Therefore, developments of deformable lenses with stroke ranges comparable to current adaptive optics deformable mirrors are of interest. In parallel, SLMs are the technique of choice to reproduce diffractive IOLs. Developments in SLM with appropriate compromise between resolution, pixel size, fill factor and control of diffractive orders will allow expanding the field of view. In addition, strategies for controlling chromatic effects will render more realistic viewing of the natural world and mimic the effect of chromatic aberration at different distances, as well as allow simulation of achromatizing IOL designs. Advances in IOL manufacturing (ranging from freeform optics, laser printing to laser induced or refractive index changes) will likely materialize novel design concepts into physical IOLs, potentially opening avenues for personalization of correction. IOL simulators will need to map these designs into the corrective elements, most interestingly with the possibility of pre-testing customizable parameters prior to manufacturing, or in certain realizations, finetune the correction following implantation. Finally, for the simulators to produce realistic simulations of vision with prospective IOLs, they need to be binocular. Strategies leading to reducing the footprint of the simulators, so that they project the physical or simulated lens on the pupil without the need bulky projection elements, are paramount. Ideally the simulators should be wearable, so that the patient can experience vision through the simulated IOL at different distances, as determined by their gaze. Developments in Augmented /Virtual Reality can serve as inspiration, including associated technologies such as eye tracking and freeform optics, metalenses, or other strategies for miniaturization.

## Data Availability

Data underlying the results presented in this paper are not publicly available at this time but may be obtained from the authors upon reasonable request.
